# Mathematical Modeling of the Effect of Pulsed Electric Field Mode and Solution Flow Rate on Protein Fouling during Bipolar Membrane Electroacidificaiton of Caseinate Solution

**DOI:** 10.3390/membranes12020193

**Published:** 2022-02-06

**Authors:** Vladlen Nichka, Semyon Mareev, Natalia Pismenskaya, Victor Nikonenko, Laurent Bazinet

**Affiliations:** 1 Physical Chemistry Department, Membrane Institute, Kuban State University, 149 Stavropolskaya Str., 350040 Krasnodar, Russia; vladlen.nichka.1@ulaval.ca (V.N.); mareev-semyon@bk.ru (S.M.); v_nikon@mail.ru (V.N.); 2 Laboratory of Food Processing and ElectroMembrane Processes (LTAPEM), Dairy Research Center (STELA), Department of Food Sciences, Institute of Nutrition and Functional Foods (INAF), Université Laval, Quebec, QC G1V 0A6, Canada; laurent.bazinet@fsaa.ulaval.ca

**Keywords:** electrodialysis, electroacidification, bipolar ion-exchange membrane, pulsed electric field, Reynolds number, ion transfer, numerical simulation

## Abstract

A one-dimensional non-stationary model was developed for a better understanding of the protein fouling formation mechanism during electroacidification of caseinate solution using electrodialysis with bipolar membranes (EDBM) in pulsed electric field (PEF) mode. Four different PEF modes were investigated with pulse–pause durations of 10–10 s, 10–20 s, 10–33 s, 10–50 s. For each current mode 3 different flow rates were considered, corresponding to Reynolds numbers, Re, equal to 187, 374 and 560. The processes are considered in the diffusion boundary layer between the surface of the cation-exchange layer of bipolar membrane and bulk solution of the desalination compartment. The Nernst–Planck and material balance equation systems describe the ion transport. The electroneutrality condition and equilibrium chemical reactions are taken into account. The calculation results using the developed model are in qualitative agreement with the experimental data obtained during the previous experimental part of the study. It is confirmed that both the electrical PEF mode and the flow rate have a significant effect on the thickness (and mass) of the protein fouling during EDBM. Moreover, the choice of the electric current mode has the main impact on the fouling formation rate; an increase in the PEF pause duration leads to a decrease in the amount of fouling. It was shown that an increase in the PEF pause duration from 10 s to 50 s, in combination with an increase in Reynolds number (the flow rate) from 187 to 560, makes it possible to reduce synergistically the mass of protein deposits from 6 to 1.3 mg/cm^2^, which corresponds to a 78% decrease.

## 1. Introduction

Electrodialysis (ED) is a process of separation of ions in aqueous solutions under the influence of an electric potential difference using electrically charged membranes. This method is increasingly being used for the recovery of protein-based compounds from meat by-products [1] and for the separation of plant secondary metabolites, peptides, proteins, polysaccharides and other functional macromolecules from complex food-based streams [2]; moreover, coupling UF membranes with ED enables the efficient separation of proteins with a similar molecular weight, which is hard to achieve by the conventional ultrafiltration (UF) membrane [3]. ED is also used for desalting lactose mother liquor before crystallization to increase lactose yield [4] or to prepare low-lactose milk powder using coupling membrane technologies [5], as well as to utilize a salty whey UF permeate [6]. To increase the efficiency of such processes, the following methods can be used: ED with porous membranes [7]; membrane stacks containing UF membranes [3]; organic-inorganic membranes and resins [8] and/or electrodialysis with bipolar membranes (EDBM) [9].

EDBM is one of the types of ED, which combines classical ED with the specific property of bipolar membranes (BPM) to split water. BPMs consist of two layers: the anion-exchange layer and the cation-exchange layer. Water splitting takes place at the junction of these layers. This method is, or can be, used in the food industry for the deacidification and demineralization of cranberry juice [10] and milk pH adjustment [11,12]; tartaric stabilization of wine [13]; purification of organic acids from fermentation broths [14]; separation of N-methylated glycine derivative amino acids [15]; acid casein production from milk [16], etc. Casein production using EDBM is a very interesting method due to high product purity and the absence of hazardous reagents and waste generation.

The expansion of uses of EDBM has increased the interest in this technique, but has also generated several problems such as fouling phenomena of membranes [17,18]. Fouling is a deposition of organic and inorganic substances on the surface and inside the pores of membranes, which leads to the degradation of properties and limitation of membrane lifetime [19]. Fouling is an actual problem in industrial processes, since the cost of cleaning procedures and membrane replacement may vary from 40 to 50% for the electromembrane processes [18,19].

The use of pulsed electric fields (PEFs) is one of the effective ways of fouling and scaling prevention or minimization [20,21,22]. The PEF mode is a non-stationary current mode which consists of application of consecutive pulse and pause lapses of a certain duration. One of the main advantages of using PEF mode is a suppression of the concentration polarization (CP) phenomenon. CP is a process where the concentration of a component increases or decreases close to the membrane surface due to the selective transport properties of the membrane. CP negatively affects the overall process efficiency of membrane processes. When using PEF, suppression of CP leads to a decrease in water splitting [23]. CP reduction also increases current efficiency, resulting in lower energy consumption per target product [24]. Additionally, the use of PEF enhances the demineralization process rate [25], increases transfer of charged biomolecules [26] and induces a selective ion migration [27].

Hydrodynamic conditions significantly affect the technological parameters of ED [16] and can also be used to effectively minimize fouling and scaling [16,28]. Turbulence-promoting spacers and their shape can also reduce CP and enhance process productivity [29]. For example, Grossman et al. [30,31] reported that an increase in flow rate and use of spacers are effective against fouling formation.

Fouling modeling is a valuable tool for preventing or minimizing this phenomenon and for determining optimal ED conditions. Gu et al. proposed a mathematical model for the prediction of the whey fouling mass in a plate heat exchanger depending on different parameters of experiment [32]. In the paper [33], the authors developed a model for the determination of peptide fouling based on the characteristics of filtration membranes. Rejabzadeh et al. [34] used Computational Fluid Dynamic method for fouling behavior description of electroacidified soy protein extracts during UF process. Another model for fouling prediction was described in [35] during the Direct Contact Condensation process. Jaegher et al. reported a different method for the modelling of the fouling formation process. The authors used a neural differential equation for the description of colloidal fouling during ED [36]. In another paper, Jaegher et al. [37] combined a mechanistic description of the transport processes with a machine learning model using neural differential equations to describe the colloidal aggregation and attachment to the surface of the ion exchange membrane. However, a specific model for the quantitative description of the protein fouling formation process during electroacidification of a caseinate solution, as far as we know, has never been developed before.

The general objective of the study is to investigate the protein fouling formation and attachment mechanism during EDBM of sodium caseinate solution using numerical simulation. The specific objectives are to study the influence of PEF pause-duration combination as well as solution flow rate on the fouling formation and dissolution rate in order to find optimal antifouling parameters of the process.

Within the framework of this theoretical study, the process of milk electroacidification by EDBM for acid casein production is considered. However, a sodium caseinate solution is used instead of milk, which is usually treated in the food industry, since the composition of the model caseinate solution as well as the absence of casein micelles are much simpler than the composition and casein micellar structure of milk for the modeling of the EDBM process. In this case, it is possible to isolate the study of the fouling kinetics from the influences of scaling by doubly charged ions, breakage of the casein micelles and slow release of calcium and phosphate from the micelles.

## 2. Experimental Section

This research consisted of the experimental study [38] of the effect of PEF and solution flow rate on the fouling kinetics during EDBM of a model sodium caseinate solution. A schematic representation of the experimental setup and some explanations are given in Supplementary Materials (SM). During the experiment, a four-chamber micro-flow cell with two Neosepta BPM, BP-1E (Astom, Tokyo, Japan), and one Neosepta cation-exchange membrane (CEM), CMX-fg, was used. The system under study (Figure S1 in SM) consisted of three closed loops containing equal volumes (300 mL) of solutions: 20 g/L Na_2_SO_4_, 2 g/L KCl and sodium caseinate. The total protein content in the model caseinate solution was similar to milk and amounted to 0.013 mol/L. Before each experiment, the KCl salt was added to the solution to achieve an initial conductivity of 3100 µS/cm and pH of 6.5. as in skim milk. Three flow rates (7.8, 15.6 and 23.4 cm/s) were investigated, corresponding to Reynolds numbers (Re) of 187, 374 and 560, respectively. An extrusion mesh spacer for flow turbulization was installed inside the ED channels. This spacer had rhombic meshes with a filament diameter of 0.07 cm and mesh step of 0.4 cm. The critical value of the Reynolds numbers for channels with similar spaces were close to 100 [29]. Thus, the hydrodynamic regime in our study was turbulent.

**Figure 1:**
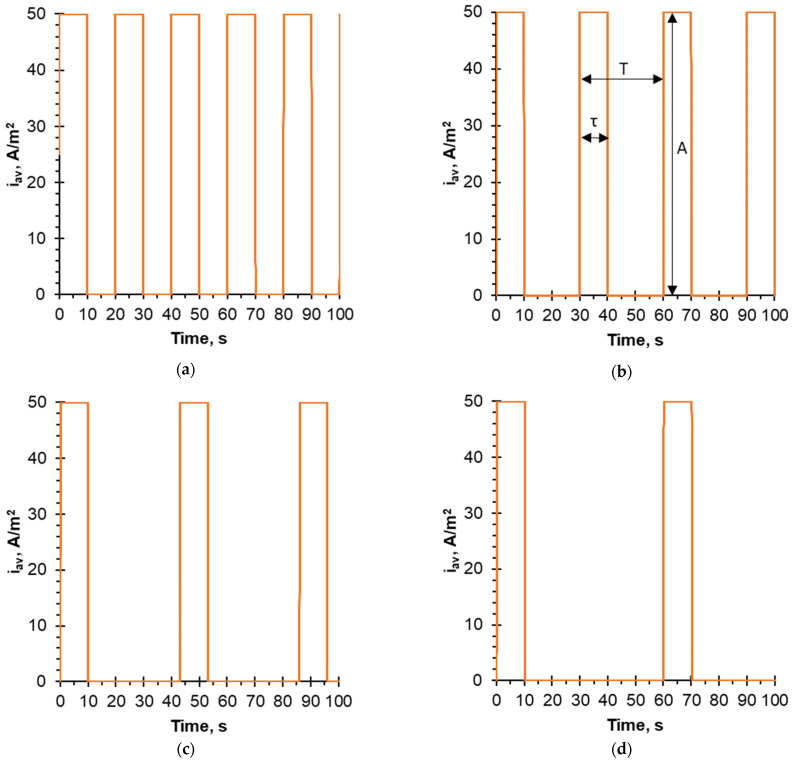
Time dependences of the current density for the PEF pulse-pause durations: (**a**) 10–10 s; (**b**) 10–20 s; (**c**) 10–33 s; (**d**) 10–50 s, where τ is the pulse duration, T is the pulse period (single pulse and single pause duration), and A is the pulse amplitude.

The PEF mode was used with a fixed pulse lapse and different pause lapse durations, namely: 10–10 s, 10–20 s, 10–33 s and 10–50 s for each flow rate. Figure 1 illustrates these PEF modes. The choice of current mode was based on the pioneering paper of Ruiz et al. [39], in which the effectiveness of the 10–40 s PEF mode was demonstrated for the first time on protein fouling mitigation and that for the same sodium caseinate solution. However, the hydrodynamic conditions and electrodialysis parameters in the experiment described in Ref. [39] were different. In addition, the optimal ratio of the duration of pulses and pauses has not been found. The maximum and minimum possible flow rates set by the pumps were used in the present experiment. In addition, an intermediate value was used to more fully describe the effect of hydrodynamic conditions. During the experiment, the durations of PEF modes were equivalent to a continuous current (CC) mode of 30 min at a current density i_av_ = 50 A/m^2^. Thus, the total process durations for the different current modes were not the same, and were equal to 60, 90, 129 and 180 min, respectively, in order to maintain the same amount of charges carried per experiment.

The experimental results showed that the fouling was formed on the cation-exchange layer of the BPM in contact with the caseinate solution in all cases considered. It has been demonstrated that both an increase in the PEF pause duration and an increase in the flow rate have a significant effect on the minimization of protein deposit (decrease by 86%), while the choice of the electric mode has the greatest effect.

## 3. Theoretical Section

In the framework of a previous experimental study [38], it was noticed that most of the protein deposit was loose and weakly attached to the BPM surface, and also easily removed from it, even with a light washing of the surface with water. However, on the surface of the cation-exchange layer of BPM, a thin, dense layer of deposit, not washable with water, was present and removable from the membrane surface only by the use of a metal spatula. Apparently, based on recent results concerning ion-exchange membrane fouling by peptides [40,41], the first dense layer of deposit on the membrane surface is formed due to electrostatic interactions of –NH_3_^+^ groups of the peptide with negatively charged –SO_3_^−^ groups of the cation-exchange layer of BPM. Subsequent loose deposit layers are formed due to hydrophobic interactions of peptides approaching the surface with an already formed dense peptide layer [42]. In addition, hydrogen bonds between oxygen-containing carboxyl groups and the hydrogen of amino groups or hydrocarbon chains of proteins can contribute to the formation of this deposit [42].

Note that proteins consist of amino acids, the structure of which can be schematically represented as ^+^H_3_N–R–COO^−^. According to the dependence of α_s_- and β- casein solubility upon the pH of sodium caseinate [38], almost all casein (from 80 to 100% depending on the fraction) is in a soluble state and has a negative charge at pH greater than 6 (Figure S2 in SM) due to –NH_3_^+^ group deprotonation (H_2_N–R–COO^−^). The negative charge of casein (indicated as Cas^−^) is caused by the presence of the –COO^−^ groups and neutral –NH_2_ groups in its structure. The acidification of the solution leads to a sharp drop in the fraction of soluble casein to zero, due to the protonation of –NH_2_ groups and formation of a neutral bipolar ions ^+^H_3_N–R–COO^−^. Thus, both fractions become neutrally charged (indicated as HCas^0^) and precipitate in the range of pH from 4.5 to 5. A further acidification leads to the appearance of a positively charged form ^+^H_3_N–R–COOH due to the protonation of negatively charged -COO^−^ groups of casein. In this case, the fraction of soluble casein re-increases. Thus, at pH 3, the fraction of soluble positively charged casein (indicated as Cas^+^) reaches 55–60% [43].

Casein charge estimates are confirmed by the ζ-potential as a function of pH dependence for β-casein. Indeed, [43] shows that ζ-potential of this casein fraction is positive at low pH (ζ-potential values are in the range from 0 to 31 mV) and has a maximum value of 31 mV at pH of 3–3.5, showing that at these pH values the highest surface charge and the strongest electrostatic interactions occur. As expected, ζ-potential of β-casein is close to 0 at pH 4.8, which is near the isoelectric point of the casein fractions and becomes negatively charged at higher pH values, up to −40 mV between pH 7 and 9. ζ-potential of β-casein in demineralized water as a function of pH at 20 °C is presented in Figure S3 in SM.

### 3.1. Problem Formulation

**Figure 2:**
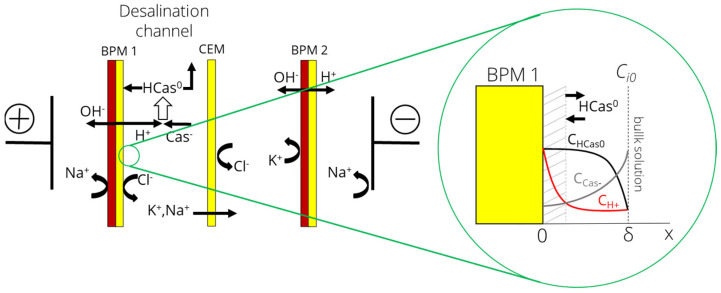
Schematic representation of the simulated system, including the BPM cationic interface (x = 0) and DBL (x = δ) of the desalination channel. Here Cas^−^ and HCas^0^ are anions and molecules of casein, respectively.

The main task of this work is to study the kinetics of protein fouling of BPM. Based on the experimental results [38], it was concluded that deposits were present only on the surface of the cation-exchange layer of BPM, and not inside the pores of the membrane; therefore, the volume of the membrane was not considered within the framework of this approach. Thus, the system under study is a diffusion boundary layer (DBL) of desalination channel bounded by the surface of the cation-exchange layer of BPM on one side and the bulk solution on the other (Figure 2).

Not all ions present in the sodium caseinate solution were taken into account due to the complexity of the mathematical calculation, but the mineral species with the highest concentration in the solution, and representing more than 80% of the total minerals, were considered in the simulation. Thus, it is assumed that there are 7 types of species: ions Na^+^, K^+^, H^+^, Cl^−^, OH^−^, caseinate anion (Cas^−^) and uncharged casein molecules (HCas^0^).

Three flow rates were investigated corresponding to the Reynolds numbers of 187, 374 and 560, used in the previous experimental work [38]. The thickness of the DBL, δ, in the model takes into account the hydrodynamic characteristics of the system. It is assumed that with an increase in the solution flow rate, the thickness of the DBL decreases. The values of the DBL thicknesses were determined from the experimental dependences δ vs. Re, presented in [44], for spacers similar to those used in the experiment [38]. They were 60 µm, 40 µm and 30 µm, respectively.

**Figure 3:**
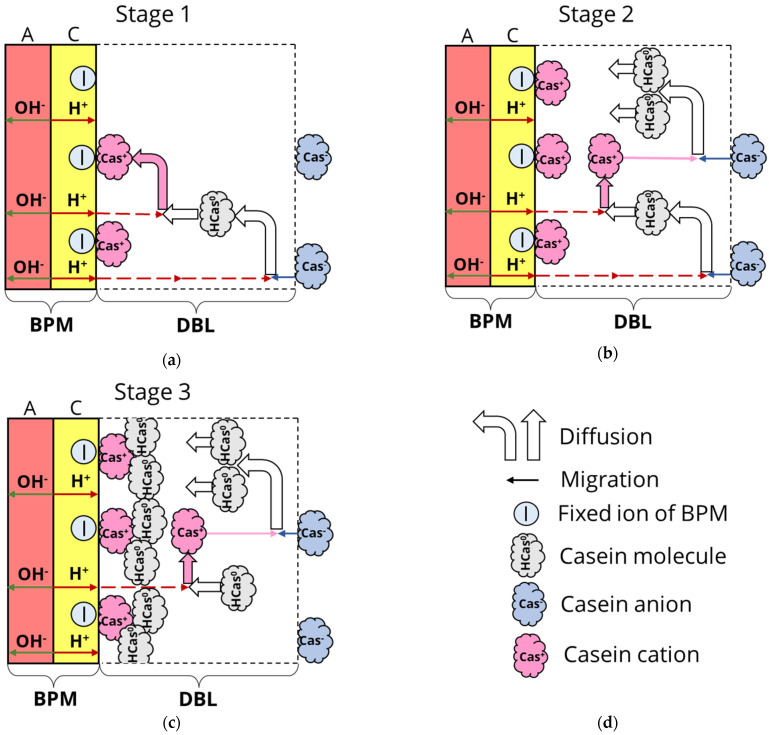
A schematic representation of the deposit formation at the cation-exchange surface of the BPM. Stage 1 (**a**): formation of casein cations (Cas^+^) caused by interactions of casein species Cas^−^ and/or HCas^0^ with protons. Stage 2 (**b**): filling the cation-exchange surface with a dense layer of casein cations; accumulation of casein molecules HCas^0^ in DBL. Stage 3 (**c**): formation of the loose deposit layer due to hydrogen bonds and hydrophobic interactions of HCas^0^ with an already formed dense deposit layer. (**d**) is a legend for this scheme. Explanations are given in the text.

Figure 3 shows a schematic representation of the deposit formation, which is based on the peptide fouling investigations reported in [40,42,45].

As mentioned in Section 3, the charge of proteins depends on the pH of the solution. In the developed model, casein anions (Cas^−^) move towards the membrane surface. The closer to the membrane surface, the lower the pH value due to the flux of H^+^ ions (Figure 3a). Therefore, as the casein molecule or ion approaches the surface, the fraction of protein molecules with a negative charge gradually decreases, while the fraction of neutrally charged molecules increases and positively charged molecules appear at the very surface of the membrane (where the value of pH is the lowest).

The concentration of casein cations Cas^+^ can be judged by the concentration of protons. Casein cations (Cas^+^) can be bound by electrostatic interactions with negatively charged functional SO_3_^−^ groups of the cation-exchange layer of BPM, as demonstrated by Persico et al. [42] in the case of cation-exchange membranes and peptides, and can also move back into the bulk solution under the action of an external electric field [46]. Moving away from the membrane surface, they acquire a neutral charge, and then a negative one, after which the electric force makes them return to the membrane surface (Figure 3b). That is, protein molecules fall into the so-called “trap” when casein molecules move from the membrane surface to the bulk solution and back depending on the acquired charge. In both cases, the effective mobility of casein cations is strongly limited. In addition to electrostatic forces, hydrophobic interactions act between protein molecules. Due to the action of these hydrophobic interactions, the uncharged protein molecules interact with the adsorbed positively charged molecules and form a loose layer at the membrane surface (Figure 3c).

Within the framework of the model, casein cations (Cas^+^) are assumed to be immobile. On the one hand, they are immobilized due to electrostatic interaction with the fixed ions of the membrane. On the other hand, they interact hydrophobically with uncharged protein molecules HCas^0^, which cannot exist in soluble form. It is assumed that the formation of a layer of uncharged protein molecules is a reversible process: this layer is formed if the HCas^0^ concentration increases with time—which occurs under the condition when current flows and H^+^ ions are generated. When the current is turned off and the generation of H^+^ ions is stopped, the pH in the DBL rises due to the diffusion of H^+^ ions from the DBL into the bulk solution. Under these conditions, the protein layer, or part of this protein layer of uncharged molecules, dissolves. The model takes into account this process, as well as the transfer of negatively charged protein molecules (Cas^−^) and the mutual transformation of negatively charged and neutral forms of the protein.

It should be noted that, during EDBM, not all protons generated by the BPM contribute to a decrease in pH of the caseinate solution, some of them are in a bound state. They can interact with casein anions (Cas^−^) to form uncharged forms that precipitate (HCas^0^) [47]:

(1) $${\mathrm{HCas}}^{0}\overset{k_{1}}{\underset{k_{-1}}{⇄}}H^{+}+{\mathrm{Cas}}^{-},$$

where $k_{1},k_{-1}$ are the forward and backward rate constants, respectively.

Thus, the reaction rate is $r_{{\mathrm{HCas}}^{0}}=-k_{1}c_{{\mathrm{HCas}}^{0}}+k_{-1}c_{H^{+}}c_{{\mathrm{Cas}}^{-}}$

Where $c_{i}$ is the concentration of ion i.

In equilibrium state: $k_{1}c_{{\mathrm{HCas}}^{0}}=k_{-1}c_{H^{+}}c_{{\mathrm{Cas}}^{-}}$

(2) $$K_{a}=\frac{k_{1}}{k_{-1}}=\frac{c_{H^{+}}c_{{\mathrm{Cas}}^{-}}}{c_{{\mathrm{HCas}}^{0}}},$$

According to the graph of protein solubility as a function of solution pH [47] the point where $c_{{\mathrm{HCas}}^{0}}=c_{{\mathrm{Cas}}^{-}}$ corresponds to $pK_{a}=\mathrm{pH}=4,8$, respectively, $K_{a}=10^{-4.8}$. Here pK_a_ is an equilibrium acid dissociation constant of the reaction (1).

For a reaction in solution k_−1_ = 10^7^ m^3^/mol s (which is equal to the water recombination constant), then $k_{1}=K_{a}k_{-1}=10^{5.2}$ s^−1^.

In addition, the system takes into account the water dissociation reaction:

(3) $$H_{2}O\overset{k_{d}}{\underset{k_{r}}{⇄}}H^{+}+{\mathrm{OH}}^{-},$$

The rate of this reaction is $r_{H_{2}O}=k_{r}c_{H^{+}}c_{{\mathrm{OH}}^{-}}-k_{d}c_{H_{2}O}$.

Then, the rates of H^+^, OH^−^, HCas^0^ and Cas^−^ formation are equal, respectively:

(4) $$R_{H^{+}}=-r_{{\mathrm{HCas}}^{0}}-r_{H_{2}O}\;R_{{\mathrm{OH}}^{-}}=-r_{H_{2}O}\;R_{{\mathrm{HCas}}^{0}}=r_{{\mathrm{HCas}}^{0}}\;R_{{\mathrm{Cas}}^{-}}=-r_{{\mathrm{HCas}}^{0}}$$

Ion transfer in the system is described by the Nernst–Planck Equation (5), the electroneutrality condition (6), as well as the material balance Equation (7):

(5) $$j_{i}=-D_{i}\left(\frac{dc_{i}}{dx}+z_{i}c_{i}\frac{F}{RT}\frac{d\varphi }{dx}\right)$$

(6) $$\sum z_{i}c_{i}=0$$

(7) $$\frac{\partial c_{i}}{\partial t}=-\frac{\partial j_{i}}{\partial x}+R_{i}$$

where $j_{i}$, $c_{i}$, $D_{i}$, $z_{i}$, are the flux density, concentration, diffusion coefficient and charge number of ion i, respectively; R is the gas constant; T is the temperature; F is the Faraday constant; φ is the electric potential.

### 3.2. Formation of Deposit Layer

It is assumed that uncharged protein molecules are formed in the bulk solution and can both diffuse to the membrane surface and be washed off by the solution flow. Thus, not all HCas^0^ molecules are deposited on a layer of absorbed positively charged molecules. When the current is turned off, all uncharged protein molecules dissolve and move into the bulk solution. The concentration of protein molecules near the membrane surface, $c_{{\mathrm{HCas}}^{0}}^{s}$, can be described by the following equation:

(8) $$\frac{\partial c_{{\mathrm{HCas}}^{0}}^{s}}{\partial t}=-ac_{{\mathrm{HCas}}^{0}}^{s}+bc_{{\mathrm{HCas}}^{0}},$$

where a and b are the dissolution and formation rate coefficients of a fouling layer, respectively. So, with an increase in a or a decrease in b coefficient, the precipitate dissolves faster, while a decrease in a, as well as an increase in the b coefficient, leads to a more rapid formation of a protein deposit.

$c_{{\mathrm{HCas}}^{0}}^{s}$ is the product of the volumetric concentration of the protein deposition, $c_{{\mathrm{HCas}}^{0}}^{0}$, on its layer thickness, α:$c_{{\mathrm{HCas}}^{0}}^{s}=\alpha c_{{\mathrm{HCas}}^{0}}^{0}$. Thus, Equation (8) can be represented as follows:

(9) $$\frac{\partial \alpha c_{{\mathrm{HCas}}^{0}}^{0}}{\partial t}=-a\alpha c_{{\mathrm{HCas}}^{0}}^{0}+bc_{{\mathrm{HCas}}^{0}},$$

Assuming that the protein concentration in the deposit layer is constant ($c_{{\mathrm{HCas}}^{0}}^{0}=const$), the following equation is obtained, in which only the deposit layer thickness changes:

(10) $$\frac{\partial \alpha }{\partial t}=-a\alpha +b\frac{c_{{\mathrm{HCas}}^{0}}}{c_{{\mathrm{HCas}}^{0}}^{0}},$$

The protein concentration in the deposit layer, $c_{{\mathrm{HCas}}^{0}}^{0}$, can be found as: $c_{{\mathrm{HCas}}^{0}}^{0}=\frac{\rho_{{\mathrm{HCas}}^{0}}}{M_{{\mathrm{HCas}}^{0}}}$, where $\rho_{{\mathrm{HCas}}^{0}}$ is the density of casein deposit ($\rho_{{\mathrm{HCas}}^{0}}$ = 1.1 g/cm^3^); $M_{{\mathrm{HCas}}^{0}}$ is the molar mass of casein ($M_{{\mathrm{HCas}}^{0}}$ = 2062 g/mol).

As already noted, it is assumed that uncharged protein molecules are formed in the bulk solution, after which they can diffuse to the membrane surface, forming a deposit layer. The formation rate of insoluble protein molecules as a function of time is described by the mass balance equation:

(11) $$\frac{\partial C_{{\mathrm{HCas}}^{0}}}{\partial t}=-\frac{\partial j_{{\mathrm{HCas}}^{0}}}{\partial x}+R_{{\mathrm{HCas}}^{0}},$$

### 3.3. Boundary Conditions

- Membrane surface

The rate of a heterogeneous reaction describing the flux of uncharged casein molecules on the membrane surface is presented as the following boundary condition:

(12) $$j_{{\mathrm{HCas}}^{0}}^{}(x=0)=R_{{\mathrm{HCas}}^{0}}^{s},$$

(13) $$R_{{\mathrm{HCas}}^{0}}^{s}=a\alpha c_{{\mathrm{HCas}}^{0}}^{0}-bc_{{\mathrm{HCas}}^{0}}^{},$$

The flux of hydrogen ions is proportional to the applied current:

(14) $$j_{H}(x=0)=\frac{it_{H}}{z_{H}F},$$

where i is the current density and $t_{H}$ the transport number in cation exchange layer of BPM. It is assumed that all the current is spent on the generation of H^+^ ions ($t_{H}=1$).

During the modeling, the PEF modes were applied, identical to those described in the experimental work [38]; namely, the pulse-pause ratios of PEF: 10–10 s, 10–20 s, 10–33 s and 10–50 s. It is assumed that during the pause of PEF, the current density is zero (i_av_ = 0), while during the pulse lapse, the current density is equal to 5 mA/cm^2^.

The potential is assumed constant and equal to zero at the BPM surface:

(15) φ(x=0)=0,

- Bulk solution (x = δ)

The concentration of every species is assumed constant and time independent:

(16) $$c_{i}(x=\delta )=c_{i0},$$

where $c_{i0}$ is the concentration of ion *i* in the bulk solution.

### 3.4. Parameters of the Model

The Cas^−^ concentration is determined using the initial casein concentration in the model solution (0.013 mol/L or 13 mol/m^3^). It is assumed that, initially, all of the casein ions in the model solution are negatively charged based on its pH value (pH = 6.5). The bulk concentration of H^+^ and OH^−^ ions is determined using the known initial pH of the solution (pH = 6.5). The bulk concentration of Na^+^ is determined from the electroneutrality equation, which is close to the value determined by Inductively Coupled Plasma-Optical Emission Spectrometry (ICP-OES) analysis [38]. The concentrations of K^+^ and Cl^−^ are calculated using Kohlrausch’s Law [48] from the known value of the initial conductivity of the experimental model solution (3100 µS/cm).

The developed model makes it possible to calculate the concentrations and fluxes of all components in the system, as well as the rate of formation and dissolution of protein deposits. All calculations were performed using the input parameters shown in Table 1.

The diffusion coefficients of Na^+^, H^+^, OH^−^, K^+^ and Cl^−^ are known values, while the diffusion coefficient of Cas^−^ was determined from the Stokes–Einstein equation:

(17) $$D_{{\mathrm{Cas}}^{-}}=\frac{k_{B}T}{6\pi \eta r},$$

where $D_{{\mathrm{Cas}}^{-}}$ is the diffusion coefficient of Cas^−^; $k_{B}$ is Boltzmann’s constant; η is the dynamic viscosity; r is the radius of the spherical particle.

It should be noted that the value of the diffusion coefficient of the uncharged protein, $D_{{\mathrm{HCas}}^{0}}$, affects the thickness of the protein deposit. It is difficult to estimate the size of the formed protein particles in order to accurately determine their radius. However, the analysis shows that the thickness of the deposit layer α increases with decreasing $D_{{\mathrm{HCas}}^{0}}$. Indeed, a decrease in the diffusion coefficient is equivalent to an increase in the thickness of the DBL (or a decrease in the flow rate in the system), which leads to an increase in the thickness of the protein deposit.

## 4. Results and Discussion

### 4.1. Evolution of pH

**Figure 4:**
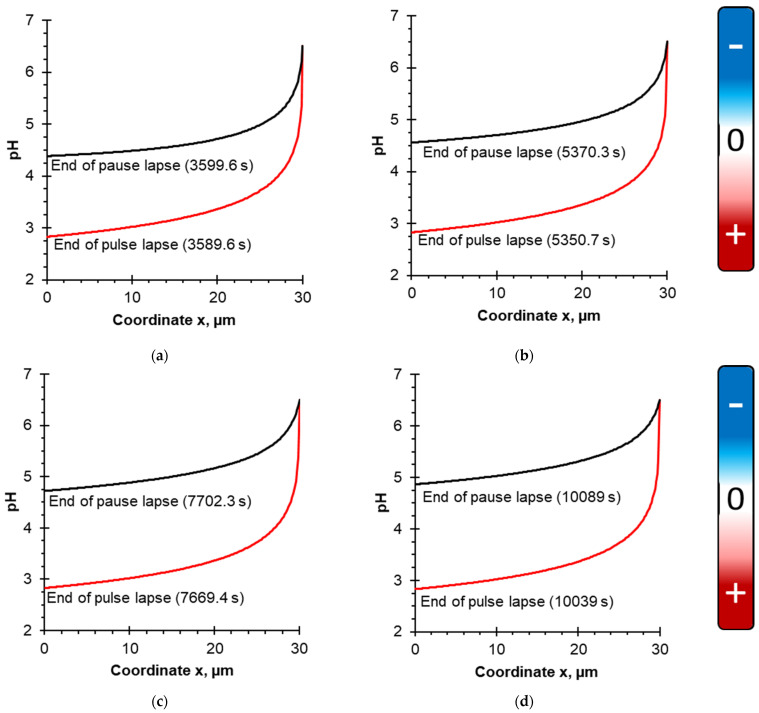
pH evolution of the caseinate solution vs the coordinate. Calculations are performed for δ = 30 μm and the following PEF modes: (**a**) 10–10 s at the end of the pulse lapse (3589.6 s) and pause lapse (3599.6 s); (**b**) 10–20 s at the end of the pulse lapse (5350.7 s) and pause lapse (5370.3 s); (**c**) 10–33 s at the end of the pulse lapse (7669.4 s) and pause lapse (7702.3 s); (**d**) 10–50 s at the end of the pulse lapse (10,039 s) and pause lapse (10,089 s). The steady-state condition is considered. To the right of the figures, a diagram of the dependence of the casein species charge on pH is given. It is based on the dependence of the zeta potential values of casein on pH [43] (Figure S3 in SM).

Figure 4 shows calculated dependences of the solution pH upon the coordinate for four considered PEF modes: 10–10 s (Figure 4a), 10–20 s (Figure 4b), 10–33 s (Figure 4c) and 10–50 s (Figure 4d). Simulations were performed at the end of the PEF pulse lapse and pause lapse; the solution flow rate corresponds to δ = 30 μm (Re = 560), which is the highest flow rate of those considered. The steady-state condition is considered. Coordinate x = 0 corresponds to the BPM surface, x = 30 μm is the outer boundary of the DBL.

According to the boundary condition of the model, the pH value in the bulk solution was set as constant (pH 6.5), because based on the experimental data [38], the pH changes in the bulk solution (in the external reservoir where the pH of caseinate was measured) were insufficient for protein precipitation (pH varied from 6.5 to 6.2). Approaching the membrane surface, the pH value decreases due to the generation of H^+^ ions by the BPM, which migrate through the cation-exchange layer and enter the DBL, acidifying it [49].

According to calculations (Figure 4), it appears that at the end of the pulse lapse for all considered PEF modes, the pH value drops to 2.8, while at the end of the pause lapse, the pH reaches different values depending on the PEF mode. An increase in the pause duration of PEF leads to an increase in pH at the membrane surface. So, in the case of the shortest pause duration (10–10 s PEF regime, Figure 4a), the pH of the solution drops to a value of 4.4, and in the case of the longest pause duration (10–50 s PEF regime, Figure 4d), the pH drops to 4.9. This effect can be explained by the fact that during the pause of PEF, the current is zero and there is no generation of H^+^ ions. Moreover, hydrogen ions diffuse from the DBL to the bulk solution during the pause lapse of PEF [27].

**Figure 5:**
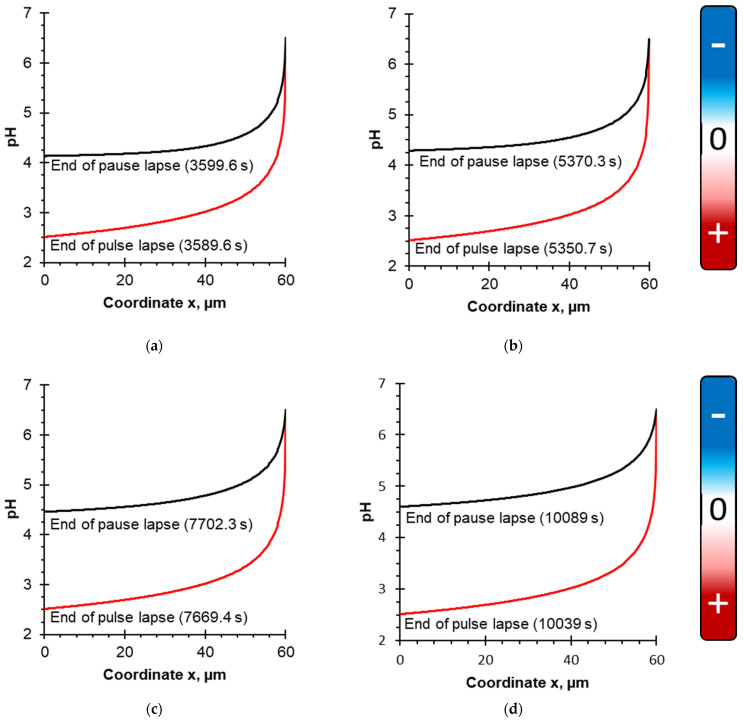
pH evolution of the caseinate solution vs the coordinate. Calculations are performed for δ = 60 μm and the following PEF modes: (**a**) 10–10 s at the end of the pulse lapse (3589.6 s) and pause lapse (3599.6 s); (**b**) 10–20 s at the end of the pulse lapse (5350.7 s) and pause lapse (5370.3 s); (**c**) 10–33 s at the end of the pulse lapse (7669.4 s) and pause lapse (7702.3 s); (**d**) 10–50 s at the end of the pulse lapse (10,039 s) and pause lapse (10,089 s). The steady-state condition is considered. To the right of the figures, a diagram of the dependence of the charge of casein species on pH is given. It is based on the dependence of the zeta potential values of casein on pH [43] (Figure S3 in SM).

Similar calculations as in Figure 4 were made for the lowest solution flow rate corresponding to Re = 187 and δ = 60 µm. As expected, the pH values of the solution at the cation-exchange layer of BPM surface become lower with a decrease in the solution flow rate (Figure 5). The pH value drops to 2.5 at the end of the pulse lapse for all considered PEF modes. At the end of pause lapse, the pH reaches values 4.1 (10–10 s PEF mode, Figure 5a) and 4.6 (10–50 s PEF mode, Figure 5d). A decrease in solution flow rate leads to less mixing of the solution. A reduction of solution mixing causes a decrease in pH values due to a deceleration in proton transport from the surface of the cation-exchange layer of BPM into the bulk solution.

### 4.2. Evolution of Species Concentration

**Figure 6:**
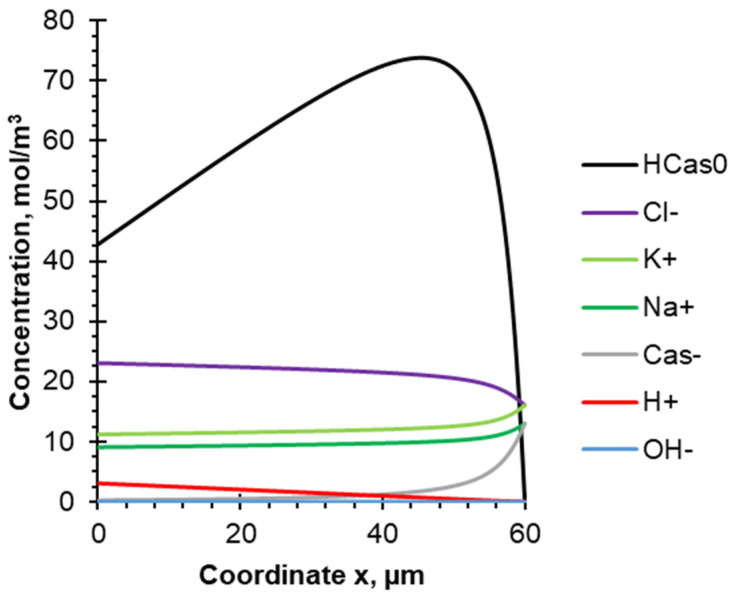
Concentration profiles of the species present in the model solution between the surface of the BPM cation-exchange layer (x = 0) and the outer boundary of the DBL (x = 60 μm). Calculations are made for the CC mode, steady state condition (1800 s after the current was turned on) and flow rate corresponding to Re = 560 and DBL thickness δ = 60 μm.

Figure 6 presents the concentration profiles of the model solution components, calculated for the CC mode, steady state (1800 s after the current was turned on) and flow rate corresponding to Re = 560 and DBL thickness δ = 60 μm. As already noted, the whole current is spent on the generation of H^+^ ions, which leave the membrane volume and interact with casein anions Cas^−^, leading to the formation of concentration profiles of anions and uncharged casein molecules HCas^0^. As a result, the concentration of Cas^−^ decreases, and the concentration of the HCas^0^ species increases in the direction from the outer boundary of the DBL to the surface of the cation-exchange layer of BPM. The maximum values of the HCas^0^ concentration (more than 70 mol/m^3^ against 13 mol/m^3^ of Cas^−^ in bulk solution) are achieved in the DBL at a distance of about 46 μm from the surface of the cation-exchange layer of BPM. This means that most of the HCas^0^ molecules are formed on the right part of the DBL (near the bulk solution), due to relatively high concentrations of both initial components (Cas^−^ and H^+^) and their participation caused by the interactions illustrated in Figure 3. The concentration of casein species increases several times compared to the bulk solution due to the so-called "trap" [50], when casein molecules move from the membrane surface to the bulk solution and back depending on the acquired charge. Moreover, the facilitated transfer of protons in the form of Cas^+^ cations to the outer boundary of the DBL and their interaction with casein anions (Figure 3b,c) to some extent resembles the active transport of substances across the cytoplasmic membrane (a sodium-potassium pump, for example), which is carried out against the concentration gradient [51]. Part of the formed casein molecules can participate, which leads to membrane fouling. The other part of the HCas^0^ moves into the bulk solution. As a result, the HCas^0^ concentration decreases as it approaches the membrane surface. This concentration reaches values of about 40 mol/m^3^ at x = 0. 

An unobvious calculation result is a decrease in the concentration of cations (K^+^, Na^+^), despite the fact that their transport numbers in the membrane are equal to zero. This is due to the H^+^ generation, which are competitors to other cations. At the same time, the Cas^−^ concentration decreases sharply and the Cl^−^ concentration increases to maintain the electroneutrality of the solution.

**Figure 7:**
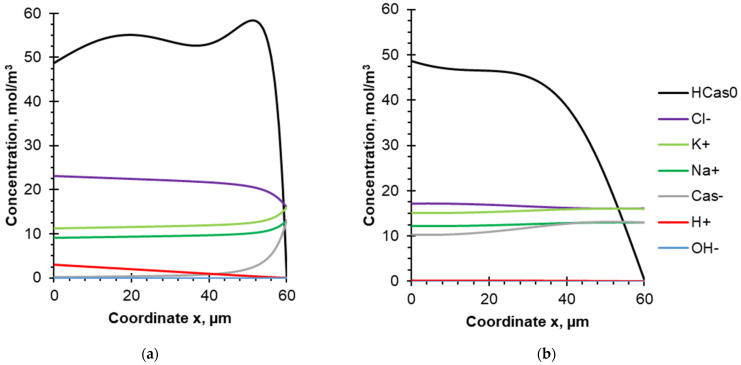
Concentration profiles of the species present in the model solution between the surface of the BPM cation-exchange layer (x = 0) and the outer boundary of the DBL (x = 60 μm). Calculations are made for the PEF mode 10–10 s at: (**a**) the end of the pulse lapse (3568.8 s after the current was turned on); (**b**) the end of the pause lapse (3579.1 s after the current was turned on). The steady-state condition and flow rate corresponding to Re = 187 and δ = 60 µm are considered.

In the case of 10–10 s PEF mode the generation of H^+^ ions and their interaction with Cas^−^ anions leads to an increase in the concentration of casein molecules HCas^0^ in the DBL during the pulse lapse (Figure 7a). At the same time, the concentration of Cas^−^ anions becomes negligible in the distance between x = 0 and x = 46 μm. It is worth noting that the HCas^0^ concentration does not increase as significantly as in the case of the CC mode near the outer boundary of the DBL, due to the short duration of the current pulse (10 s). Moreover, this concentration remains practically constant in the DBL. A slight decrease (up to 50 mol/m^3^), indicating the participation of HCas^0^ in the deposit layer formation, occurs only at a distance from 0 to 10 μm.

When the current is turned off, the H^+^ concentration drops to zero (Figure 7b). The uncharged form of protein begins to dissolve due to the pH increase, and therefore the concentration of HCas^0^ decreases. Thus, casein acquires a negative charge and the concentration of Cas^−^ increases. It is also worth noting that the concentration of HCas^0^ is higher near the membrane surface and decreases towards the bulk solution up to complete dissolving. Since the current is turned off only for 10 s, the concentration profile of HCas^0^ does not have enough time to form completely; then the current is turned on again, and the generation of H^+^ continues. This explains the curved shape of the concentration profile of the uncharged protein HCas^0^, which is not observed in the case of CC superposition (Figure 6).

It should be noted that during pulse lapse of PEF mode, the oscillating concentration profile of HCas^0^ is observed (Figure 7a). A closer look explains their nature. When the current is turned off (during pause lapse of PEF), the system tends to an equilibrium state. Concentrations of K^+^, Na^+^, Cas^−^ and OH^−^ increase, while Cl^−^ and H^+^ concentrations decrease. The diffusion coefficients of the mentioned ions (except Cas^−^) are in the order of 10^−9^ m^2^/s, and after 10 s their concentration profiles are approaching the equilibrium state. The Cas^−^ concentration also approaches the equilibrium state due to the electromigration component of the flow induced by the other ions of the electric field, as well as by the chemical reaction Equation (1). HCas^0^ partially dissociates and transforms into the Cas^−^ form. Dissolution of the fouling layer formed on the membrane surface increases the HCas^0^ concentration. As can be seen from Figure 7b, after 10 s, the maximum value of the concentration of the casein molecule is observed at the membrane surface (where the protein deposit dissolves), and the minimum value is in the bulk solution. The diffusion coefficient of HCas^0^ is several times lower than that of the other ions present in the solution, which leads to a slower decrease in its concentration, and it remains relatively high in the middle of the DBL.

After switching on the current (pulse lapse of PEF), H^+^ ions generated by the BPM move into the DBL and react with Cas^−^. The HCas^0^ concentration increases sharply near the membrane surface. After the moment when all Cas^−^ ions have reacted with H^+^ near the membrane surface, the region of the HCas^0^ formation shifts closer to the bulk solution. A deposit forms on the surface of the cation exchange layer of the BPM, and the HCas^0^ concentration decreases slightly. In the middle of the DBL, the concentration of HCas^0^ increases slowly. As a result, 10 s after switching on the current, an oscillating concentration profile is observed with maximums in the regions of HCas^0^ formation near the membrane surface (immediately after switching on the current, when the concentration of Cas^−^ is still high) and close to the bulk solution (where the concentration of Cas^−^ is constantly high).

**Figure 8:**
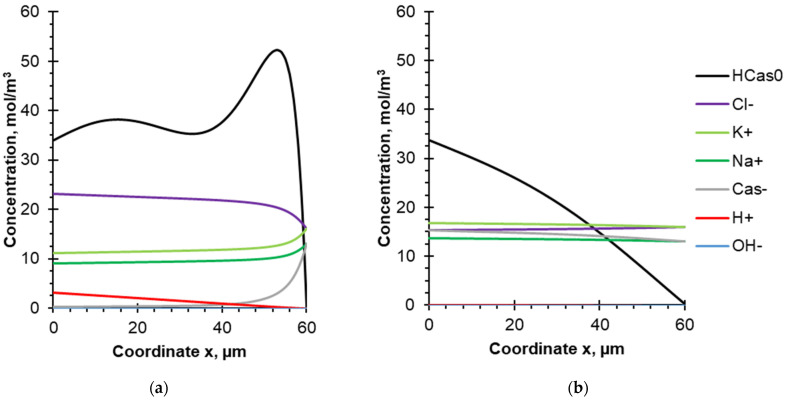
Concentration profiles of the components of the model solution between the surface of the BPM cation-exchange layer (x = 0) and the outer boundary of the DBL (x = 60 μm). Calculations are made for the PEF mode 10–33 s in: (**a**) the end of the pulse lapse (7669 s after the current was turned on); (**b**) the end of the pause lapse (7701.7 s after the current was turned on). The steady-state condition and flow rate corresponding to Re = 187 and δ = 60 µm are considered.

The concentration profiles at the end of the pulse lapse (Figure 8a) and the pause lapse (Figure 8b) for the 10–33 s PEF mode are presented to estimate the effect of the PEF pause duration on the concentration distribution of the different species. The concentration of uncharged protein molecules in this case does not grow as fast as in the case of the 10–10 s PEF mode (Figure 7). In addition, it can be seen from Figure 8b that the concentration of protein molecules decreases more significantly with an increase in the PEF pause duration, which confirms the positive antifouling effect with an increase in the PEF pause duration, described in the experimental section of the work. Indeed, during the pause lapse of PEF, a relaxation of the concentration profile occurs in the DBL. The species concentrations return partially or completely to their bulk solution values, which causes partial dissolving of the protein deposit layer.

### 4.3. Quantification of Fouling

**Figure 9:**
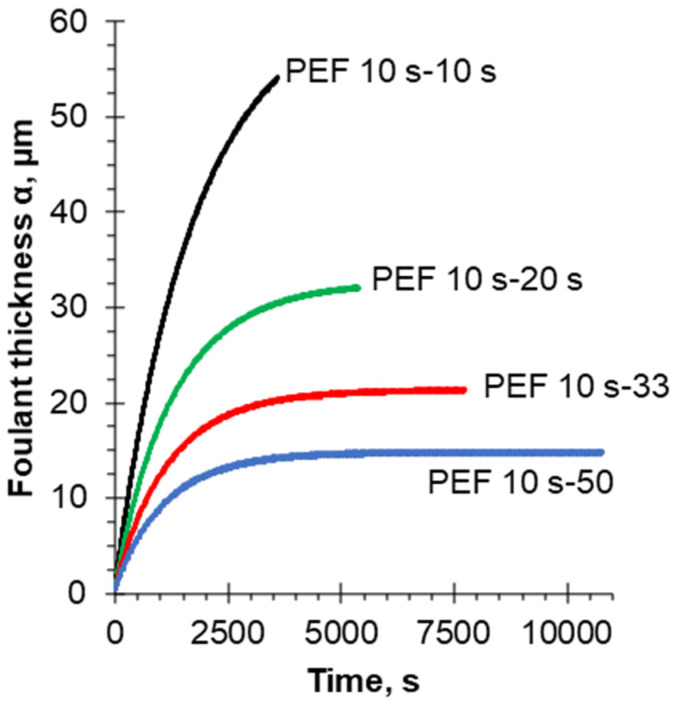
Time dependence of the protein deposit layer thickness at various current modes. Calculations are made for the steady-state condition and flow rate corresponding to Re = 374 and δ = 40 µm.

Figure 9 shows that the thickness of the protein deposit layer (foulant), α, in all current modes gradually increases during the experiment, reaching a stationary value in the case of using the PEF modes of 10–33 s and 10–50 s with a DBL thickness δ = 40 μm, which corresponds to Re = 374. In the case of a shorter pause duration, namely in the PEF modes of 10–10 s and 10–20 s, the selected experimental durations are not enough to achieve a stationary state. It can be seen that when using a PEF, the thickness of the deposit is smaller the longer the PEF pause duration is. Thus, the use of the 10–20 s PEF pulse-pause mode allows reduction of the deposit thickness as compared to the 10–10 s PEF mode, from 53.9 to 31.9 μm, which corresponds to a 41% decrease.

It should be noted that the thickness of the protein fouling increases during each pulse lapse and decreases during pauses, which confirms the assumption about the formation of a deposit during each PEF pulse and dissolution during pauses. Indeed, during the PEF pause, there is no generation of H^+^ ions, while the flow of the solution intensifies the diffusion of hydrogen ions from the DBL into the bulk solution. In addition, the solution flow can partially wash off the protein deposit from the membrane surface. The thickness of the protein deposit in the 10–33 s PEF mode is 21.2 μm. The most effective PEF mode among the considered ones in the modeling process turned out to be the 10–50 s PEF mode, which is consistent with the experimental data reported earlier [38]. In this case, the thickness of the deposit by the end of the process was 14.7 μm.

Note that the experiment [38] with a 10–100 s PEF mode demonstrated no significant difference in deposit mass compared to the 10–50 s PEF mode. In addition, the use of the 10–100 s PEF mode increased the duration of the experiment up to 5 h, which caused a significant evaporation of the treated solutions. Therefore, the 10–50 s PEF mode seems to be the most optimal among all the studied modes.

**Figure 10:**
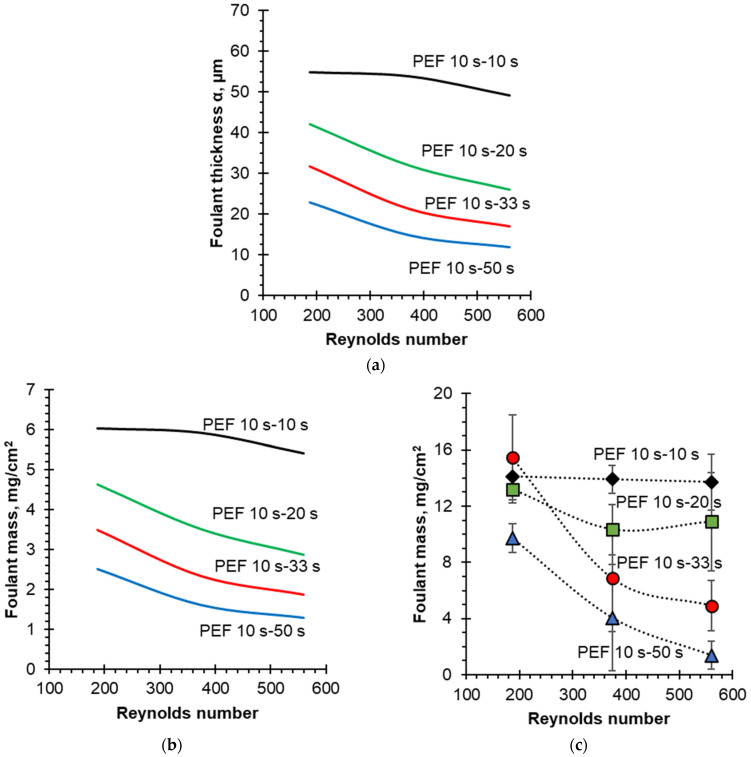
The calculated thickness (**a**) and the mass (**b**) as well as the experimental mass (**c**) of the protein deposit layer vs. the Reynolds number for different PEF modes. The mass of protein is normalized to the active membrane surface area.

Figure 10a shows a dependence of the thickness of the protein deposit layer (foulant) as a function of the Reynolds numbers calculated for different PEF modes. In addition, a transition from the thickness of the protein deposit layers to their mass was made (Figure 10b), provided that the density of the deposit is known (ρ = 1.1 g/cm^3^). For a correct comparison of theoretical and experimental data, the mass of protein deposit must be normalized per unit of the active surface area of the membrane (the surface exposed to the electrical current passage). The active surface of the membrane was 10 cm^2^; therefore, the protein deposit mass was normalized to this value. The data obtained confirms that both the choice of the PEF mode and the flow rate have a significant effect on the thickness (and, accordingly, the mass) of the protein deposit during EDBM of sodium caseinate solution. Moreover, the main contribution is made by the choice of the electric current mode, and an increase in the PEF pause duration leads to a decrease in the thickness of the protein deposit layer. This result agrees with the experimental data presented in the article [38]. Hence, an increase in the PEF pause duration from 10 s to 50 s, in combination with an increase in the solution flow rate from Re = 187 to Re = 560, make it possible to reduce the fouling mass from 6 to 1.3 mg/cm^2^, which corresponds to a 78% decrease. Thus, the calculation results (Figure 10) are in qualitative agreement with the experimental data where a decrease of 86% was observed.

However, the results of foulant mass normalized on the active membrane surface area showed differences between the experimental and the simulation values. The mass of the protein deposit removed from the membrane surface after the experiment was from 1.4 to 15.5 mg/cm^2^ depending on the experimental conditions applied (Figure 10c), while in the case of the simulation, the range of protein deposit was from 1.3 to 6 mg/cm^2^ (Figure 10b). This difference can be explained by the fact that during the experiment [38] a spacer was placed in the intermembrane space. In this case, apart from the tangential component (along the membrane surface), there is a normal component (perpendicular to the membrane surface) of the convective ion flux to the membrane surface. In addition, the normal component is directed both towards the membrane surface and away from it, bending around the spacer filaments. Within the framework of the approach used in this paper, the contribution of the spacer is taken into account indirectly, only through the DBL thickness. This may explain the difference in the amount of protein deposit between the experimental data and the calculated results.

## 5. Conclusions

A one-dimensional non-stationary model was proposed here taking into account the equilibrium protonation-deprotonation reactions of casein and allowing to estimate the pH of the solution in the DBL at the cation-exchange surface of a BPM as a function of the distance from the membrane surface, the current mode and the flow rate.

The use of the PEF mode and an increase in the flow rate of the solution reduce by several times the thickness and weight of protein deposit on the cation-exchange surface of BPM. The greatest positive antifouling effect is achieved with a current pulse duration of 10 s and a pause of 50 s in combination with Reynolds number of 560. The synergistic combination of optimal current and hydrodynamic modes reduces the mass of casein deposits on the surface of the BPM by 78% compared to the 10–10 s PEF mode in combination with Reynolds number of 187. These simulation results are in qualitative agreement with the experimental data obtained under the same conditions.

The simulation allows a better understanding of the mechanism of casein deposit formation on the BPM cation-exchange surface. During the PEF pause, there is no current flow and, accordingly, there is no generation of H^+^ ions. At this time, the concentration profiles are partially restored, reducing the concentration polarization. Diffusion of H^+^ ions into the bulk solution causes a pH increase in DBL. This explains the dissolution of fouling during pause lapse of PEF and, accordingly, the effect of its duration on the thickness of the deposit.

Increasing the solution flow rate and, consequently, a decrease in DBL thickness is similar to the use of PEF. It consists of reducing the concentration polarization.

The next step is the modification of the developed model in order to describe quantitatively the experimental results of the fouling in the EDBM process of caseinate-containing solutions.

## Figures and Tables

**Table 1:** Main input parameters used in the calculation.

Symbol	Value	Description
δ	60, 40, 30 µm ^1^	DBL thicknesses
$i_{av}$	5 mA/cm^2^	Average current density
$D_{{\mathrm{Na}}^{+}}$	1.33 × 10^−9^ m^2^/cm	Ion (molecule) diffusion coefficients in the solution
$D_{H^{+}}$	9.3 × 10^−9^ m^2^/cm	
$D_{{\mathrm{OH}}^{-}}$	5.27 × 10^−9^ m^2^/cm	
$D_{K^{+}}$	1.98 × 10^−9^ m^2^/cm	
$D_{Cl^{-}}$	2.03 × 10^−9^ m^2^/cm	
$D_{{\mathrm{Cas}}^{-}}$	6.6 × 10^−11^ m^2^/cm	
$D_{{\mathrm{HCas}}^{0}}$	1 × 10^−12^ m^2^/cm	
$k_{1}$	1.58 1/s	Forward rate constant of the reaction (1)
$k_{-1}$	1 × 10^7^ m^3^/s mol	Backward rate constant of the reaction (1)
$K_{a}$	0.016 mol/m^3^	Equilibrium acid dissociation constant of the reaction (1)
a	1 1/s	Dissolution rate coefficient of the fouling layer
b	5 × 10^−4^ m/s	Formation rate coefficient of the fouling layer
pH^0^	6.5	The initial pH value of the solution

^1^ DBL thicknesses are equal to 60, 40 and 30 µm, which corresponds to Reynolds numbers 187, 374 and 560, respectively. These values were based on the results presented in [44].

## Supplementary Materials

The following are available online at www.mdpi.com/article/10.3390/membranes12020193/s1, Figure S1: Configuration of ED cell for caseinate solution eletroacidification by EDBM, Figure S2: Solubility of αS-casein and β-casein in sodium caseinate solution at 20 °C, Figure S3: ζ-Potential of β-casein in demineralized water as a function of pH at 20 °C.

## Nomenclature

BPM	Bipolar membrane
CC	Continuous current
CEM	Cation-exchange membrane
CP	Concentration polarization
DBL	Diffusion boundary layer
ED	Electrodialysis
EDBM	Electrodialysis with bipolar membranes
ICP-OES	Inductively coupled plasma-optical emission spectrometry
PEF	Pulsed electric field
SM	Supplementary material
UF	Ultrafiltration
