# Supplementary material for: Mathematical Modeling of the Effect of Pulsed Electric Field Mode and Solution Flow Rate on Protein Fouling during Bipolar Membrane Electroacidificaiton of Caseinate Solution
Source: Membranes (Basel). 2022 Feb 6;12(2):193. doi: 10.3390/membranes12020193 (PMC8877438; doi:10.3390/membranes12020193)
Supplement: Supplementary file 1 [file membranes-12-00193-s001.zip › membranes-1549073-supplementary.pdf]

Mathematical Modeling of the Effect of Pulsed Electric Field Mode and Solution Flow Rate on Protein Fouling during Bipolar Membrane Electroacidificaiton of Caseinate Solution

Vladlen Nichka [1,2], Semyon Mareev [1], Natalia Pismenskaya [1,*], Victor Nikonenko [1] and Laurent Bazinet [2]

[1] Membrane Institute, Kuban State University, 149 Stavropolskaya Str., 350040 Krasnodar, Russia; mareev-semyon@bk.ru (S.M.); v_nikon@mail.ru (V.N.)
[2] Laboratoire de Transformation Alimentaire et Procédés ÉlectroMembranaires (LTAPEM, Laboratory of Food Processing and ElectroMembrane Processes), Dairy Research Center (STELA), Department of Food Sciences, Institute of Nutrition and Functional Foods (INAF), Université Laval, Québec, QC G1V 0A6, Canada; vladlen.nichka.1@ulaval.ca (V.N.); laurent.bazinet@fsaa.ulaval.ca (L.B.)
* Correspondence: n_pismen@mail.ru (N.P.)

Supplementary materials

1. Experimental Part

This research is based on the experimental study of the effect of PEF and solution flow rate on the fouling kinetics during EDBM of a model sodium caseinate solution [1].

Casein sodium salt (or sodium caseinate) used in the experimental study was purchased from Sigma Chemical Co. (St. Louis, MO, USA). Na_2SO_4 (ACS grade) and KCl (ACS grade) were obtained from BDH (VWR International Inc., Mississauga, ON, Canada). Chemicals for cleaning-in-place of the ED system (HCl and NaOH) were purchased from Fisher Scientific (Montreal, QC, Canada). The caseinate powder was reconstituted with distilled water in order to have the same content of casein as in skim milk. Thus, the total protein content in the model caseinate solution is 27 g/L. Solution was held overnight under gentle agitation at 10 C to allow its complete solubilization and rehydration. Moreover, potassium chloride salt was added in the model solution before each experiment in order to have the same initial conductivity as for skim milk (about 3100 μs/cm).

Casein sodium salt is made from casein of bovine milk, the main protein presented in milk. Casein, which makes up approximately 80% of the total protein in bovine milk, is presented in four forms: α-s1 Casein, α-s2 Casein, β-Casein and κ-Casein. The approximate casein composition of milk is (g/L): α-s1 (12–15); α-s2 (3–4); β (9–11); and κ (2–4). The casein subunits vary primarily in molecular weight, isoelectric point, and level of phosphorylation.

During the experiment, a four-chamber micro-flow cell (Electro-Cell AB, Täby, Sweden) with two Neosepta BPM (BP-1E) and one Neosepta CEM (CMX-fg) was used (Figure 1S). The active membrane surface was 10 cm_2. The system under study consisted of three closed loops containing equal volumes (300 ml) of solutions: 20 g/L Na_2SO_4, 2 g/L KCl and sodium caseinate. During the experiment, three flow rates (7.8, 15.6 and 23.4 cm/s) were investigated, corresponding to Reynolds numbers (Re) of 187, 374 and 560, respectively. The flow rate was set the same for all solutions. Each closed loop was connected to an external reservoir for continuous recirculation of the solutions.

The variation in temperature during the whole process was about 12 °C (from 25 °C to 37 °C). This variation was the same for each mode of current. In the current study, the ED system was not equipped to maintain a constant temperature. Controlling the temperature during ED process would use lots of energy, and the possible energy saving related to the fouling mitigation would be lost. Thus, to be closer to the reality and to test all experimental parameters in real conditions, the temperature increase was consequently not controlled.

Extrusion mesh - spacers for flow turbulization were installed inside the ED channels. These spacers had rhombic meshes with a filament diameter of 0.07 cm and mesh step of 0.4 cm. The critical value of the Reynolds numbers for channels with similar spaces is close to 100 according to the literature data [2]. Thus, the solution flow regime in our studies was turbulent.

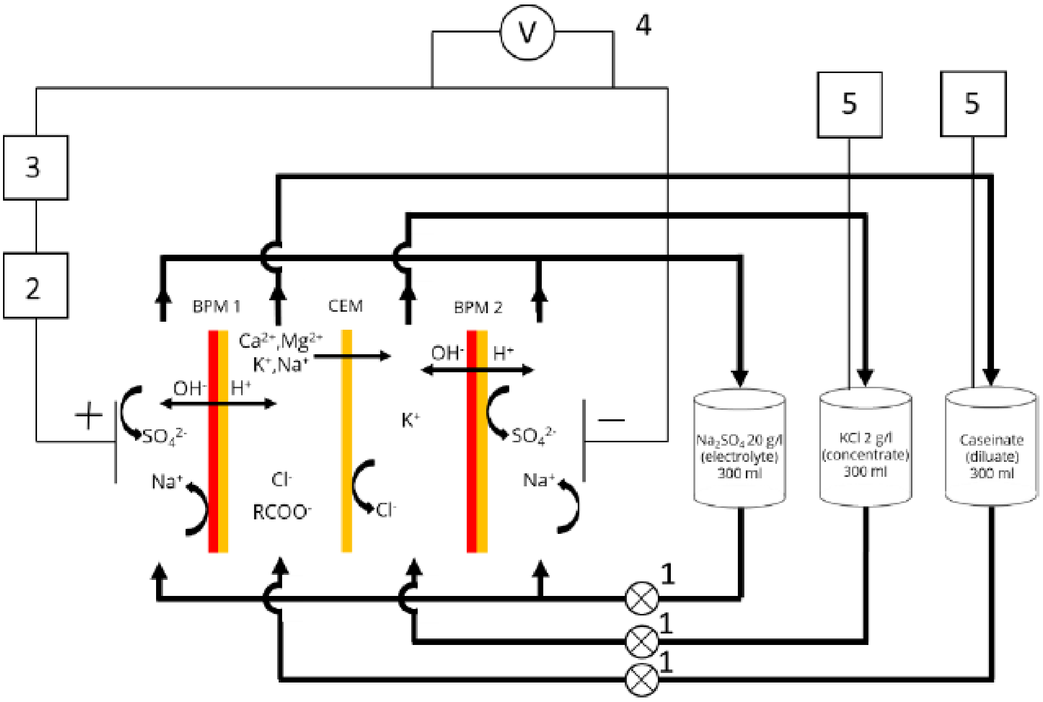

Figure S1. Configuration of ED cell for caseinate solution eletroacidification by EDBM: Electric pump (1); Power supply (2); PEF generator (3); Voltmeter (4); Measurement cells connected with pH meter and Conductometer (5). Adapted from [1].

To study the combined effect of hydrodynamic conditions and electric current mode on the fouling kinetics, the PEF mode was used with a fixed pulse lapse and different pause lapse durations, namely: 10 s-10 s, 10 s-20 s, 10 s-33 s and 10 s-50 s. In addition, one complementary experiment with 10 s–100 s PEF ratio was carried out to test whether a significant increase in the PEF pause time would affect the fouling kinetics. EDBM was carried out using a constant current intensity of 50 mA (corresponding to a current density of 3.13 mA/cm^2) generated by using a Xantrex power supply, model HPD 60-5SX (Xantrex Technology Inc., Burnaby, BC, Canada). PEF was generated by a modified Pulsewave 760 generator from Bio-Rad laboratories (Richmond, BC, Canada). The durations of PEF modes were equivalent to a continuous current mode of 30 minutes at a current density i_{av}= 50 A/m^2. Thus, the total process durations for the different current modes were not the same and equal to 60, 90, 129 and 180 minutes, respectively, in order to maintain the same amount of charge carried per experiment. Three replicates of each combination (flow rate–PEF regime) were performed (36 experiments total).

The conductivity of model caseinate and KCl solutions passing through the cell compartments was measured with an YSI conductivity meter Model 3100 with an YSI immersion probe Model 3252, cell constant K = 0.1 cm^{-1} (Yellow Springs Instrument Co., Yellowsprings, OH, USA). A pH-meter Model SP70P from VWR International (Montreal, QC, Canada) was used to measure the pH of caseinate solution. The pH of KCl solution was measured with an Orion Star A221 pH-meter from Thermo Scientific (Montreal, QC, Canada).

Protein fouling was collected from the membrane surfaces right after ED cell dismounting, and then freeze-dried in a Labconco lyophyliser, Model Freezone 4.5 (Kansas City, MO, USA). After weighing, the dried powder samples were kept at -20 °C until further analysis.

The experimental results showed that the fouling was formed on the cation-exchange layer of the BPM contacted with the caseinate solution. It has been demonstrated that both an increase in the PEF pause duration and an increase in the flow rate have a significant effect on the minimization of protein deposit (decrease by 86%), while the choice of the electric mode has the greatest effect.

2. Effect of pH on Casein Charge

In the framework of a previous experimental study [1], it was noticed that most of the protein deposit was loose and weakly attached to the BPM surface, and also easily removed from it, even with a light washing of the surface with water. However, on the surface of the cation-exchange layer

of BPM, a thin dense layer of deposit, not washable with water, was present and removable from the membrane surface only by the use of a metal spatula. Apparently, based on recent results concerning ion-exchange membrane fouling by peptides [3,4], the first layer of deposit on the membrane surface is formed due to electrostatic interactions of –NH_{3+} groups of the protein with negatively charged –SO_{3-} groups of the cation-exchange layer of the BPM. Subsequent loose fouling layers are formed due to hydrogen bonds and hydrophobic interactions of proteins approaching the surface with an already formed dense protein layer [5].

According to the dependence of α_s- and β- casein solubility upon the pH of sodium caseinate (Figure 2S) [6], almost all casein (from 80 to 100% depending on the fraction) is in a soluble state and has a negative charge at pH greater than 6 (Figure 2S). The negative charge of casein is caused by the presence of the – COO_- group in its structure. Further acidification of the solution leads to a sharp drop in the fraction of soluble casein to zero, due to the protonation of –COO_- groups and formation of a neutral –COOH form.

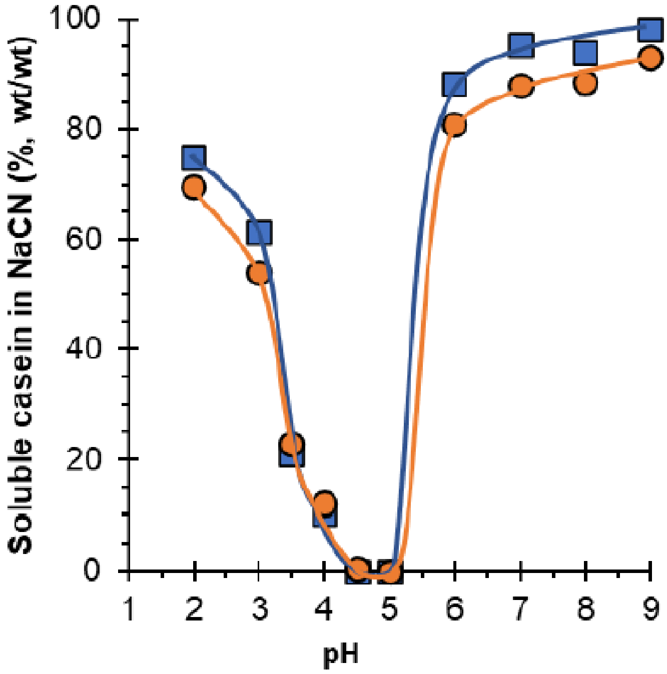

Figure S2. Solubility of αs-casein (blue curve) and β-casein (orange curve) in sodium caseinate solution at 20°C. Adapted from [6].

Thus, both fractions become neutrally charged and precipitate in the range of pH from 4.5 to 5. A further acidification leads to the appearance of a positively charged form of –NH_{3+} due to the protonation of uncharged –NH_2 groups of casein. In this case, the fraction of soluble casein reincreases. Thus, at pH 3, the fraction of soluble positively charged casein reaches 55-60 % (Figure 2S).

Casein charge estimates are confirmed by the ζ-potential as a function of pH dependence for β-casein (Figure 3S). Indeed, Figure 3S shows that ζ-potential of this casein fraction is positive at low pH (ζ-potential values are in the range from 0 to 31 mV) and has a maximum value of 31 mV at pH of 3-3.5, showing that at these pH values, the highest surface charge and the strongest electrostatic interactions occur. As expected, ζ-potential of β-casein is close to 0 at pH 4.8, which is near the isoelectric point of the casein fractions, and becomes negatively charged at higher pH values, up to -40 mV between pH 7 and 9.

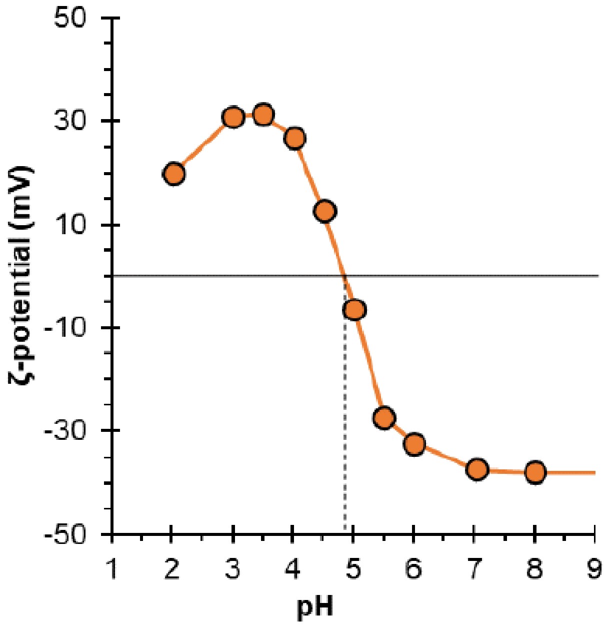

Figure 3S. ζ-Potential of β-casein in demineralized water as a function of pH at 20°C. Adapted from [6].

References

1. Nichka, V.S.; Nikonenko, V. V.; Bazinet, L. Fouling mitigation by optimizing flow rate and pulsed electric field during bipolar membrane electroacidification of caseinate solution. *Membranes (Basel).* **2021**, *11*, doi:10.3390/membranes11070534.

2. Balster, J.; Pünt, I.; Stamatialis, D.F.; Wessling, M. Multi-layer spacer geometries with improved mass transport. *J. Memb. Sci.* **2006**, *282*, 351–361, doi:10.1016/j.memsci.2006.05.039.

3. Persico, M.; Bazinet, L. Fouling prevention of peptides from a tryptic whey hydrolysate during electromembrane processes by use of monovalent ion permselective membranes. *J. Memb. Sci.* **2018**, *549*, 486–494, doi:10.1016/j.memsci.2017.12.021.

4. Abou-Diab, M.; Thibodeau, J.; Deracinois, B.; Flahaut, C.; Fliss, I.; Dhulster, P.; Nedjar, N.; Bazinet, L. Bovine hemoglobin enzymatic hydrolysis by a new ecoefficient process—part i: Feasibility of electrodialysis with bipolar membrane and production of neokyotophin (α137-141). *Membranes (Basel).* **2020**, *10*, 1–22, doi:10.3390/membranes10100257.

5. Persico, M.; Mikhaylin, S.; Doyen, A.; Firdaous, L.; Hammami, R.; Chevalier, M.; Flahaut, C.; Dhulster, P.; Bazinet, L. Formation of peptide layers and adsorption mechanisms on a negatively charged cation-exchange membrane. *J. Colloid Interface Sci.* **2017**, *508*, 488–499, doi:10.1016/j.jcis.2017.08.029.

6. Post, A.E.; Arnold, B.; Weiss, J.; Hinrichs, J. Effect of temperature and pH on the solubility of caseins: Environmental influences on the dissociation of α S- and β-casein. *J. Dairy Sci.* **2012**, *95*, 1603–1616, doi:10.3168/jds.2011-4641.
